# Resistance training-induced dihydrotestosterone is blunted in aging rat skeletal muscle

**DOI:** 10.1016/j.jphyss.2025.100019

**Published:** 2025-03-27

**Authors:** Yung-Li Hung, Akihito Ishigami, Shuichi Machida

**Affiliations:** a Japan Society for the Promotion of Science, 5-3-1 Kojimachi, Chiyoda-ku, Tokyo 102-0083, Japan; b Graduate School of Health and Sports Science, Juntendo University, 1–1 Hirakagakuendai, Inzai, Chiba 270-1695, Japan; c Molecular Regulation of Aging, Tokyo Metropolitan Institute for Geriatrics and Gerontology, 35–2 Sakae-cho, Itabashi-ku, Tokyo 173-0015, Japan; d Institute of Health and Sports Science & Medicine, Juntendo University,1–1 Hirakagakuendai, Inzai, Chiba 270-1695, Japan

**Keywords:** Resistance training, Steroidogenesis, Muscle hypertrophy, Aging

## Abstract

This study aimed to investigate the influence of aging on steroid hormone production in skeletal muscles in response to resistance training. Male F344 rats, aged 4 months (young) and 22 months (old), were randomized into the sedentary and training groups. The training group performed resistance training by climbing a ladder with a load every three days for eight weeks. After the training period, the flexor hallucis longus muscle was dissection, and muscle steroid hormone levels were analyzed using liquid chromatography-tandem mass spectrometry. We found that resistance training significantly increased muscle mass in young and old rats, although the increase was less pronounced in the latter. In young, trained rats, muscle dihydrotestosterone levels were approximately 35-fold higher compared to sedentary controls (p < 0.01); dihydrotestosterone levels did not differ significantly between sedentary and trained old rats. These findings indicate that resistance training-induced dihydrotestosterone production is blunted in aging rat skeletal muscle.

## 1 Introduction

Steroid sex hormones, secreted by tissues including the reproductive organs, liver, kidney, brain, and skeletal muscles [1], play a crucial role in regulating various physiological functions such as energy metabolism and skeletal muscle hypertrophy [2], [3]. Aging is associated with a significant decline in steroid sex hormone levels, which impairs metabolic regulation and negatively affects overall health [4]. The aging-induced reduction in steroid sex hormones may lead to muscle atrophy [5]. While the aging-related decline in steroid sex hormone production, particularly in Leydig cells, is well documented, the effects of aging on steroidogenesis in skeletal muscle remain poorly understood.

Steroidogenesis in skeletal muscle involves the conversion of dehydroepiandrosterone (DHEA), a precursor of androgens, into androstenedione (A-dione) by the enzyme 3β-hydroxysteroid dehydrogenase (HSD), and subsequently into testosterone by 17β-HSD. Testosterone is then converted into the potent androgen dihydrotestosterone (DHT) by the enzyme 5α-reductase [1]. Furthermore, administration of DHT induced the mTOR signaling pathway and muscle hypertrophy [6]. Skeletal muscle steroidogenesis can be stimulated by mechanical stress, such as endurance and resistance exercise, which has been shown to increase both muscle and serum steroid concentrations [7], [8]. Notably, resistance training increases both muscle steroid hormone production and muscle hypertrophy and is an important method for the prevention of sarcopenia [8]. Administration of steroid hormones enhances resistance-induced muscle hypertrophy in older men [9], [10]. In contrast, Welle et al. demonstrated that aging attenuates muscle hypertrophy in response to resistance training [11]. However, the direct relationship between exercise-induced muscle steroid hormone production and muscle hypertrophy is still debated.

Previous research has demonstrated that young men have higher blood testosterone levels compared to middle-aged men following resistance training, suggesting that age influences the hormonal response to exercise [12]. However, the specific effects of aging on resistance training-induced changes in muscle steroid hormone levels have not been extensively studied. Most existing studies focused on systemic hormone levels, leaving a gap in the understanding of local steroidogenesis in skeletal muscle. Sato et al. indicated that muscle steroidogenesis was increased in older men following resistance training [8], but did not compare it with resistance training-induce muscle steroidogenesis in young men. However, the effects of aging on steroid hormone production in response to resistance training are unclear. We hypothesized that aging would result in the attenuation of muscle steroid hormone production in response to resistance training. Therefore, this study aimed to investigate the effects of aging on muscle steroid hormone production in response to resistance training. Aging influences muscle adaptation to exercise at the molecular level, which may have important implications for health and physical function of older adults.

## 2 Methods

### 2.1 Experimental animals

Thirteen 4-month-old and eighteen 22-month-old male F344 rats were purchased from Charles River Laboratories Japan, Inc. (Yokohama, Japan). The animals were housed under controlled environmental conditions (23 ± 1 °C, 55 ± 5 % relative humidity) under a 12-h/12-h light/dark cycle. The rats were provided ad libitum access to water and a standard laboratory diet. The animals were randomly divided into the following groups: 4-month-old rats were assigned to the sedentary group (6 mo-Sed, n = 7) or climbing training group (6 mo-CT, n = 6), and 22-month-old rats were assigned to the sedentary group (24 mo-Sed, n = 9) or climbing training group (24 mo-CT, n = 9). All experimental procedures were conducted in accordance with the ethical guidelines for animal experiments. The study was approved by the Committee for Animal Experiments at Sakura Campus, Juntendo University (S3).

### 2.2 Ladder climbing training

Ladder climbing was chosen as the exercise activity as it effectively mimics resistance training in rats, promoting muscle hypertrophy and strength gains. The climbing training protocol was adapted from a previously established method [13], [14]. Rats in the training groups climbed a 1-meter-long ladder inclined at 85°, with rungs spaced 2 cm apart. Training sessions were conducted every 3 days, with a total of 20 sessions for 8 weeks. Rats were acclimated to the ladder without loading for 2 days prior to the first session. During the first session, the first 4 ladder climbs consisted of carrying a load equal to 50 %, 75 %, 90 %, and 100 % of the rat’s body weight. The climb was repeated after 2 min of rest. After the first 4 ladder climbs, the loads were increased by 30 g per ladder climb up to 10 ladder climbs. The load was progressively reduced as the rats completed more ladder climbs due to the increasing difficulty in completing the ladder climb task with this load. The maximal load that each rat successfully carried to the top of the ladder was recorded as it’s carrying capacity in that session. For training sessions 2–20, the first 4 ladder climbs consisted of 50 %, 75 %, 90 %, and 100 % of the maximum load in the previous session, respectively. Thereafter, the load was increased by 30 g for each ladder climb, and was progressively reduced as the rats completed more ladder climbs due to the increasing difficulty in completing the ladder climb task with this load.

### 2.3 Muscle and plasma sampling

The flexor hallucis longus (FHL) is the primary mover during ladder climbing movement [13], [14], [15]. Therefore, the FHL muscle is subject to major mechanical stress and hypertrophic stimulation after ladder climbing training compared with the other leg muscles [15]. Forty-eight hours after the final training session, the FHL muscles from both legs were dissected, weighed, and either frozen in liquid nitrogen for biochemical analysis or in isopentane cooled by liquid nitrogen for immunohistochemical analysis. Plasma was obtained by centrifugation of blood samples at 3000 rpm for 10 min at 4 °C. All muscle and plasma samples were stored at −80 °C until further analysis.

### 2.4 Immunohistochemistry

The medial belly region of the FHL muscle was subjected to myosin heavy chain (MHC) typing to measure the mean muscle fiber cross-sectional area (CSA) and muscle fiber-specific CSA. Frozen cross-Section (10 µm) were stained with mouse monoclonal antibodies against MHC I, IIa, and IIx, and rabbit polyclonal antibodies against laminin. Briefly, muscle sections were fixed in 4 % paraformaldehyde in phosphate-buffered saline (PBS) and permeabilized using 1 % Triton X-100 in PBS. After blocking with 10 % normal goat serum (NGS) at 25 °C for 1 h, the sections were incubated with primary antibodies at 25 °C for 1 h. Primary antibodies against MHC I (clone BA-F8) (1:100), anti-MHC IIa (clone SC-71) (1:100), anti-MHC IIx (6H-1) (1:100) (Developmental Studies Hybridoma Bank, Coralville, IA, USA), and anti-laminin (Sigma Aldrich, St. Louis, MO, USA) (1:100) were diluted in PBS containing 5 % NGS. After washing with PBS, the sections were incubated for 1 h at 25 °C with secondary antibodies (goat anti-mouse IgG [Invitrogen, Carlsbad, CA, USA] for BA-F8 and SC-71, goat anti-mouse IgM [Invitrogen] for 6H-1, and goat anti-rabbit IgG [Invitrogen] for laminin). Secondary antibodies were diluted to 1:500 in PBS containing 5 % NGS. Images of stained sections were obtained using a fluorescence microscope (BZ-X800; Keyence, Osaka, Japan). The CSA of approximately 600 fibers/muscle sections was calculated using the Keyence analyzer software. Furthermore, Type IIb muscle fibers are sparse in the FHL of male rats, and were not detected in the training groups in our previous studies [13], [14].

### 2.5 Quantification of muscle and plasma steroid hormone

Before sample preparation, the rat skeletal muscle was homogenized in 1 mL of distilled water using Ultra-Turrax homogenizer. As internal standards, A-dione-^13^C_3_, T-^13^C_3_, DHT-^13^C_3_, DHEA-^13^C_3_ were added to the rat skeletal muscle suspension. The steroids were extracted from each matrix with 4 mL of methyl *tert*-butyl ether. After the organic layer was evaporated to dryness, the extract was dissolved in 0.5 mL of methanol and diluted with 1 mL of distilled water. The sample was applied to the Oasis MAX cartridge, which had been successively conditioned with 3 mL each of methanol and distilled water. After the cartridge was washed with 1 mL each of distilled water, methanol/distilled water/acetic acid (45:55:1, v/v/v), and 1 % pyridine solution, the steroids were eluted with 1 mL of methanol/pyridine (100:1, v/v). After evaporation, the residue was subjected to derivatization as described below.

The sample was reacted with 50 µL of mixed solution (80 mg of 2-methyl-6-nitrobenzoic anhydride, 20 mg of 4-dimethylaminopyridine, and 40 mg of picolinic acid in 1 mL of acetonitrile) and 10 µL of triethylamine for 30 min at 25 °C. After the reaction, the sample was dissolved in 0.5 mL of ethyl acetate/hexane/acetic acid (15:35:1, v/v) and the mixture was applied to the InertSep SI cartridge, which had been successively conditioned with 3 mL each of acetone and hexane. The cartridge was washed with 3 mL of hexane and then the underivatized steroid (A-dione) was eluted with 2.5 mL of methyl *tert*-butyl ether. After elution with the underivatized steroids, picolynyl ester steroids (testosterone, DHT and DHEA) were eluted with 2.5 mL of acetone/hexane (7:3, v/v). After evaporation, the residue (picolynyl ester steroids) was dissolved in 0.1 mL of acetonitrile/distilled water (2:3, v/v) and 20 µL of the solution was subjected liquid chromatography-tandem mass spectrometry (LC-MS/MS). The underivatized steroids were further reacted with 0.1 mL of 20 mg of ethoxyamine hydrochloride in 0.1 mL of 60 % acetonitrile solution at 60 °C for 25 min. After reaction, 10 µL of the sample was subjected to LC-MS/MS, as described below.

An API-5000 triple stage quadrupole mass spectrometer equipped with a positive electrospray ionization source and an Agilent1290 Infinity LC system was employed. N_2_ was used as the collision gas. The other conditions of measurement for each steroid were as follows. A mobile phase consisting of 0.1 % formic acid and acetonitrile was used with gradient elution. Xpress C18 column (2 µm, 2.1 × 100 mm) was used at 50° for the measurement of testosterone, DHT, and DHEA. Acquity UPLC HSS C18 SB (1.8 µm, 2.1 ×150 mm) was used at 45 °C for a measurement of A-dione. The SRM transitions of A-dione, A-dione-^13^C_3_, testosterone, T-^13^C_3_, DHT, DHT-^13^C_3_, DHEA and DHEA-^13^C_3_ were *m/z* 373.4/274.3, 376.4/277.3, 394.2/253.2, 397.2/256.2, 396.3/255.1, and 399.3/203.2, 394.2/253.0 and 397.2/256.2, respectively. The limits of quantification of A-dione, testosterone, DHT, and DHEA were 0.25, 0.5, 0.5 and 0.5 pg/tube, respectively.

### 2.6 Statistical analysis

All data were expressed as the mean ± standard deviation. Statistical analysis was performed using the linear mixed model two-way analysis of variance, followed by the Bonferroni post-hoc test, using GraphPad Prism 9 software (GraphPad, Inc., San Diego, CA, USA). Statistical significance was set at p < 0.05.

## 3 Results

### 3.1 Muscle hypertrophy induced by resistance training is reduced in aging male rats

**Fig. 1:**
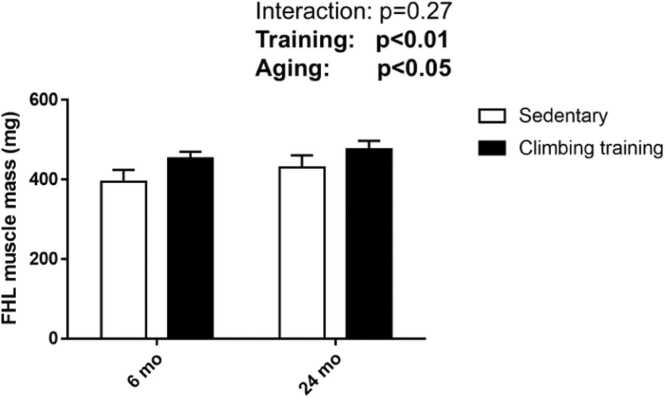
Effects of climbing training on muscle mass. Values are presented as the mean ± SD (n = 6–9). 6 mo Sed, 6-month-old sedentary; 6 mo CT, 6-month-old climbing training; 24 mo Sed, 24-month-old sedentary; 24 mo CT, 24-month-old climbing training; SD, standard deviation.

**Fig. 2:**
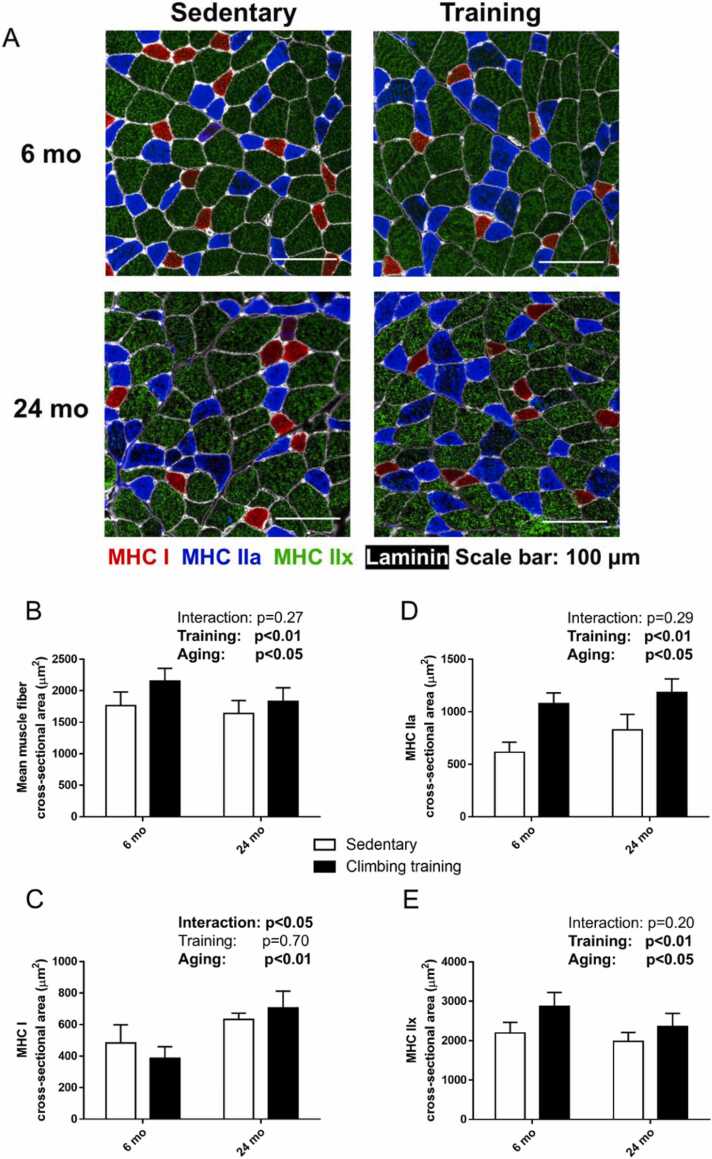
Effects of climbing training on the cross-sectional area of the flexor hallucis longus (FHL) muscle. **(A)** Representative immunohistochemical staining of MHC. The reactivity of major fiber types, including type Ⅰ (red), type IIa (blue), and type IIx (green), is shown for FHL muscles. **(B–E)** Cross-sectional area of the FHL muscle. **(B)** Mean muscle fiber cross-sectional area, **(C)** MHC I, **(D)** MHC IIa, and **(E)** MHC IIx. Values are presented as the mean ± SD (n = 6). MHC, myosin heavy chain; 6 mo Sed, 6-month-old sedentary; 6 mo CT, 6-month-old climbing training; 24 mo Sed, 24-month-old sedentary; 24 mo CT, 24-month-old climbing training; SD, standard deviation.

Following resistance training, muscle mass increased by 14.9 % in the 6-month-old male rats and by 10.6 % in the 24-month-old male rats, relative to their respective sedentary controls (main effect of training, p < 0.01; Fig. 1). The mean muscle fiber CSA in the 6-month-old and 24-month-old rats increased significantly by 22.1 % and 11.8 %, respectively, compared to the age-matched sedentary groups (main effect of training, p < 0.01; Fig. 2B). The CSA of type I muscle fiber did not show significant changes in response to resistance training (main effect of training, p = 0.73) (Fig. 2C). In contrast, the CSA of type IIa muscle fibers increased substantially in both age groups, with a 75.3 % increase in 6-month-old rats and 43.1 % increase in 24-month-old rats compared to their sedentary counterparts (main effect of training, p < 0.01; Fig. 2D). Similarly, the CSA of type IIx muscle fibers increased by 30.6 % in the 6-month-old rats and by 18.5 % in 24-month-old male rats following training (main effect of training, p < 0.01; Fig. 2E).

### 3.2 Age-related differences in muscle DHT levels following resistance training

**Fig. 3:**
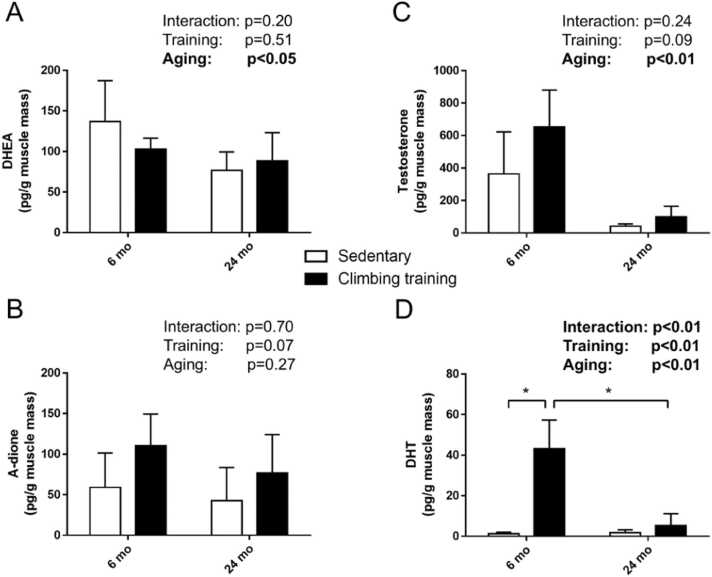
Effects of aging on resistance training-induced muscle steroidogenesis. Muscle levels of **(A)** DHEA, **(B)** A-dione, **(C)** testosterone, and **(D)** DHT. Values are presented as the mean ± SD (n = 3–4). * : p < 0.05, Tukey post-hoc test. 6 mo Sed, 6-month-old sedentary; 6 mo CT, 6-month-old climbing training; 24 mo Sed, 24-month-old sedentary; 24 mo CT, 24-month-old climbing training; SD, standard deviation; DHT, dihydrotestosterone; DHEA, dehydroepiandrosterone; A-dione: androstenedione.

While resistance training did not significantly alter DHEA and testosterone levels compared to the sedentary groups, aging resulted in a marked decrease in these hormones (main effect of aging, p < 0.05; Fig. 3A and C). The muscle A-dione levels remained unaffected by both training and aging (Fig. 3B). Interestingly, muscle DHT levels increased by approximately 35-fold in the young rats following resistance training compared to the young sedentary controls (p < 0.01), whereas no significant difference in DHT levels was observed between the old sedentary and training groups (Fig. 3D).

### 3.3 Plasma hormonal changes following resistance training

**Fig. 4:**
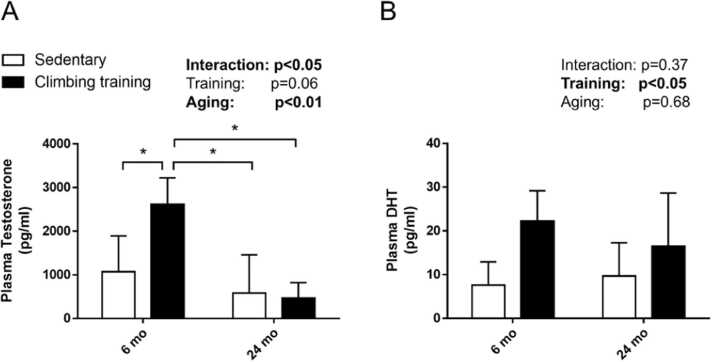
Effects of aging on plasma steroid hormones following resistance training. Plasma levels of **(A)** testosterone and **(B)** DHT. Values are presented as the mean ± SD (n = 3–4). * : p < 0.05, Tukey post-hoc test. 6 mo Sed, 6-month-old sedentary; 6 mo CT, 6-month-old climbing training; 24 mo Sed, 24-month-old sedentary; 24 mo CT, 24-month-old climbing training; SD, standard deviation; DHT, dihydrotestosterone.

In plasma, testosterone levels were significantly elevated in the young rats subjected to resistance training compared to the sedentary controls (p < 0.01). However, no such increase was evident in older rats, with no significant difference between the old sedentary and old training groups (Fig. 4A). Both the 6-month-old and 24-month-old rats in the resistance training group had higher plasma DHT levels than those in the age-matched sedentary groups (main effect of training, p < 0.05; Fig. 4B).

## 4 Discussion

This study investigated the effects of resistance training on muscle hypertrophy and steroidogenesis in aging male rats. Thus, the resistance training animal model critically contributes to investigate this issue. The ladder climbing training is closely related to human progressive resistance training and is less stressful for animals. Furthermore, ladder climbing training is a replicable method that induces effective muscle hypertrophy [15], [16]. The findings show that both 6-month-old and 24-month-old rats experienced increases in muscle mass following resistance training; however, this increase was significantly attenuated in the older rats. Additionally, the resistance training-induced increases in mean muscle fiber CSA and type IIx CSA were more pronounced in younger rats. These findings align with those of Mero et al. who reported a greater increase in muscle fiber CSA and type II fibers in younger men (26.0 ± 4.3 years old) compared to older men (61.2 ± 4.1 years old) after 21 weeks of resistance training [17]. These results suggest that aging reduces the rate of resistance training-induced muscle hypertrophy.

In terms of steroidogenesis, resistance training did not significantly affect DHEA and testosterone levels in the FHL muscle of either group. However, DHT levels in the FHL muscle rose significantly in younger rats following training, whereas no such increase was observed in older rats. In contrast, previous studies in humans have shown that 12 weeks of resistance training significantly increased DHT levels in older men (age: 67.2 ± 1.8 years) [8]. The discrepancy between animal and human muscle responses suggests that muscle-specific factors may influence steroidogenic adaptations to resistance training. These findings support the notion that resistance training-induced DHT may differ across species, warranting further investigation. Furthermore, Sato et al. analyzed muscle steroid hormones using the enzyme-linked immunosorbent assay (ELISA), and not LC-MS/MS [8]. The ELISA cannot directly detect steroid hormones, which limits its ability to measure multiple steroid hormones per sample. The LC-MS/MS, which was used in this study, is the gold standard for the direct detection of the concentrations of multiple classes of hormones and capable of highly sensitive and specific measurement [18].

Plasma testosterone levels were significantly increased in 6-month-old male rats following 8 weeks of resistance training, while no significant change was observed in 24-month-old male rats. Arazi et al. reported a similar trend, where younger men (21.2 ± 2.2 years old) showed a greater increase in plasma testosterone compared to middle-aged men (49.7 ± 2.1 years old) after 8 weeks of training [12]. Given that plasma testosterone levels are closely linked to muscle hypertrophy [19], but the direct relationship between resistance training-induced testosterone production and muscle hypertrophy is needed further investigation.

There are several limitations to this study. First, the use of a rats as a model may limit the generalizability of the findings to humans, given potential differences in muscle physiology and hormonal regulation across species. Additionally, the study focused on the FHL muscle, which may not fully represent the effects of resistance training across different muscle types. The relatively short duration of the training regimen may also not capture long-term adaptations, particularly in aging populations where muscle and hormonal responses may require more extended periods to manifest.

Overall, this study demonstrated that aging ameliorates the effects of resistance training on muscle hypertrophy, muscle DHT levels, and plasma testosterone. This study has several implications. First, it highlights the importance of considering age-related decline in steroidogenesis when designing resistance training programs for older individuals. Second, alternative strategies, such as hormone replacement therapy or modified training protocols, may be necessary to counteract the attenuated muscle and hormonal adaptations observed in older individuals. Future research should investigate the molecular mechanisms underlying the aging-related decline in DHT production in skeletal muscle. Additionally, studies should explore the differential responses of various muscle types to resistance training and examine whether alternative training regimens can mitigate the effects of aging on muscle hypertrophy and steroidogenesis.

## Abbreviations

DHEA: dehydroepiandrosterone.

A-dione: androstenedione.

DHT: dihydrotestosterone.

FHL: flexor hallucis longus.

MHC: myosin heavy chain.

CSA: cross-sectional area.

## Funding

This work was supported partly by a JSPS KAKENHI Grant Numbers 21H03296, 23KF0069 (to S.M.), and 19K19887 (Y. H.), and Institute of Health and Sports Science and Medicine, 10.13039/501100005731Juntendo University (to S.M.).

## CRediT authorship contribution statement

**Hung Yung-Li:** Writing – original draft, Investigation, Data curation, Conceptualization. **Ishigami Akihito:** Writing – review & editing, Conceptualization. **Machida Shuichi:** Writing – review & editing, Conceptualization.

## Declaration of Competing Interest

All the authors declare that they have no conflict of interest exists.

## Glossary

NA: Not applicable
